# Supramolecular chemistry: from aromatic foldamers to solution-phase supramolecular organic frameworks

**DOI:** 10.3762/bjoc.11.222

**Published:** 2015-11-02

**Authors:** Zhan-Ting Li

**Affiliations:** 1 Department of Chemistry, Fudan University, 220 Handan Road, Shanghai 200433, China

**Keywords:** donor–acceptor interaction, foldamer, hydrogen bond, radical cation dimerization, supramolecular organic framework

## Abstract

This mini-review covers the growth, education, career, and research activities of the author. In particular, the developments of various folded, helical and extended secondary structures from aromatic backbones driven by different noncovalent forces (including hydrogen bonding, donor–acceptor, solvophobicity, and dimerization of conjugated radical cations) and solution-phase supramolecular organic frameworks driven by hydrophobically initiated aromatic stacking in the cavity of cucurbit[8]uril (CB[8]) are highlighted.

## Review

### Childhood and growing up

I was born on July 23rd, 1966 in the small, remote village of Fang-Liu (a combination of two common Chinese family names), which is located in Shangcai County in the Henan Province of central China. Living in the distant countryside, my childhood was simple and quiet. Later I learned that from 1966 to 1976 China had seriously suffered from the so-called Cultural Revolution. In 1972 I entered primary school. My class had about fifteen students. We were all children from the same village. For the first two years, we had only one teacher, Xi Liu, who taught us Chinese and arithmetic. Once per week he also taught music, mainly singing. Since we did not have a permanent classroom, Mr. Liu frequently moved the class to different empty rooms that he found in the village. As I remember it, I studied in at least four “classrooms”. So every day we all needed to bring a small stool from home to “go to school”, but I did not feel this was a burden. Actually this was possibly the happiest stage of my life because Mr. Liu was a neighbor and a family friend, and my scores in Chinese and arithmetic were always the best. In addition to going to school, I also spent a lot of time reading Chinese novels that I could find after I was able to read. Although there were no opportunities for modern sports such as basketball and football, I liked playing ground chess, a two-person game that was very popular during poor times but has now nearly completely disappeared. I enjoyed the chess game very much because I could often win even against adults. However, I never dreamed that I would become a chemistry professor in Shanghai many years later.

In 1979, I entered Caigou High School, which was located in Caigou Town one kilometer away from my home village. In 1977, China reinstated the long suspended national college entrance examination, thanks to the comeback of Mr. Xiaoping Deng, the greatest Chinese leader. For young people living in the rural areas, acceptance into college was the only route to change their fate at that time. Therefore, I studied very hard and again got the highest total score in the school in the national college entrance examination in 1981. In autumn of that year, I enrolled in the Department of Chemistry at Zhengzhou University. I chose chemistry as my major only because my chemistry score was 95 (out of 100), which was higher than my math or physics scores. I had no idea on how a university picked its students, but just instinctively believed choosing chemistry meant a better chance for being admitted. After more than 30 years, I still remember the days when I studied in Caigou High School. It was quite good at that time in the county, but it could not attract teachers and students in later years due to its remote location and was closed about ten years ago by the local government.

### Studying at Zhengzhou University

Zhengzhou is the capital of the Henan Province. Being admitted to the University gave me the opportunity to leave Shangcai County and to take a train for the first time. The Chemistry Department enrolled a total of 120 undergraduates in that year. In the 1980s, the department had only the essential courses of inorganic, organic, physical and structural chemistry and chemical engineering, which were accompanied by a series of fundamental experiments in the same semester. This was the typical course system of the day in China and so all students received the same training. Many years later, China introduced the credit system and both mandatory and optional courses were offered. By the end of the third year, students were required to choose one of the above “secondary degree” disciplines. I chose organic chemistry because I felt it was the easiest course among the others. In particular, I enjoyed one course called “Organic Synthesis Skills” taught by Professor Zhixin Huang, which introduced multistep synthesis. I must say that since 1987 I have benefited greatly from this course as a graduate student at the Shanghai Institute of Organic Chemistry (SIOC). In the spring of 1985, the second semester of the fourth year, I entered Professor Zhendong Chen’s lab to perform my dissertation research. I stayed in the lab for about three months and prepared several ferrocene-derived conjugated molecules to study the homologous linearity [1]. I later realized that this was an important research project in physical organic chemistry in China in the 1980s. After graduation from Zhengzhou University, I was assigned to work at Henan Medical University as a teaching assistant. Two years later, I was enrolled as a graduate student at SIOC, the top research institution for organic chemistry in China.

### Studying at SIOC: N···I interaction

**Scheme 1:**
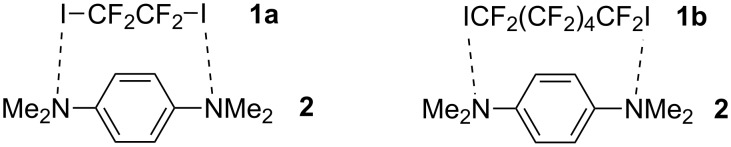
Proposed structures of complexes between **1a** and **1b** with **2**.

I joined SIOC in late August of 1987. For many years, graduate students in the first year studied physical organic chemistry, organic synthesis, and organic analytical chemistry. Impressively, all students performed 6–8 multistep synthesis experiments that involved the use of all standard organic synthesis techniques. This was a challenge to many students, but all received systematic training in organic synthesis upon completion of the experiments. In 1988, I entered Professor Ching-Sung Chi’s group in the Laboratory of Organic Fluorine Chemistry for a master’s degree. The lab is well-known for its longstanding research on reactions of fluorine-containing molecules. Professor Chi left as a visiting scholar at the University of Fribourg, Switzerland shortly after I joined the group and I was actually advised by Professor Yong-Da Lin. I finished the synthesis of several fluorine-containing macrocycles and published my first research paper in the journal Heterocycles [2]. The starting materials for these macrocycles were initially designed for the preparation of biologically active molecules, which was the main project in this laboratory. Using them to prepare new macrocycles became a small independent dissertation project for me. In 1990, I joined Professor Qing-Yun Chen’s group as a Ph.D. candidate. Professor Chen is a distinguished, esteemed Chinese chemist in organic fluorine chemistry. His group developed new trifluoromethylation reagents, which found many practical applications [3]. I respect him for his persistent passion for science. Even at the age of 86 in 2015, he still goes to his office and advises his students and remains active in the field of fluorine chemistry. I received my Ph.D. degree in December of 1992. My dissertation research focused on photo-induced reactions of perfluoroalkyl iodides and pentafluoroiodobenzene with arenes, aromatic ethers and amines and heterocycles. These reactions led to the perfluoroalkylation or pentafluorophenylation of the aromatic compounds. One series of reactions involved liquid tetrafluoro-1,2-diiodoethane (**1a**) or dodecafluoro-1,6-diiodohexane (**1b**) and solid *N*,*N*,*N*’,*N*’- tetramethylphenylene-1,4-diamine (**2**). We found that when mixing in chloroform, the 1:1 mixtures readily gave a high yield of solid adducts, which melted at 85 and 65 °C, respectively. Elemental analyses supported a 1:1 stoichiometry for the solid adducts, and ^19^F NMR spectra showed downfield chemical shifting of the IC*F*_2_ signal of **1a** and **1b** [4]. Clearly, an important intermolecular interaction occurred which led to the solidification of the mixtures [5]. However, we did not obtain the crystal structure of the mixtures, although I had been to Beijing for X-ray diffraction experiments at Peking University. We thus proposed that the two compounds formed 1:1 charge-transfer complexes (Scheme 1). Currently, this N···I interaction is termed as halogen bonding, which is widely used in supramolecular crystal engineering [6–7].

My Ph.D. training in synthetic methodology and fluorine chemistry had an important influence on my research activity. When I initiated a project, the first thing I would think of and discuss with my students is the synthetic route for the target molecules. Fluorine-containing molecules have always been my favorite. When I performed my postdoctoral research in Denmark, I used fluorine-containing precursors to build catenanes [8], and many years later, I utilized fluorine as a hydrogen bonding acceptor to develop aromatic amide and triazole foldamers [9–10].

### Postdoctoral research at the University of South Denmark: donor–acceptor interaction-driven catenanes

**Scheme 2:**
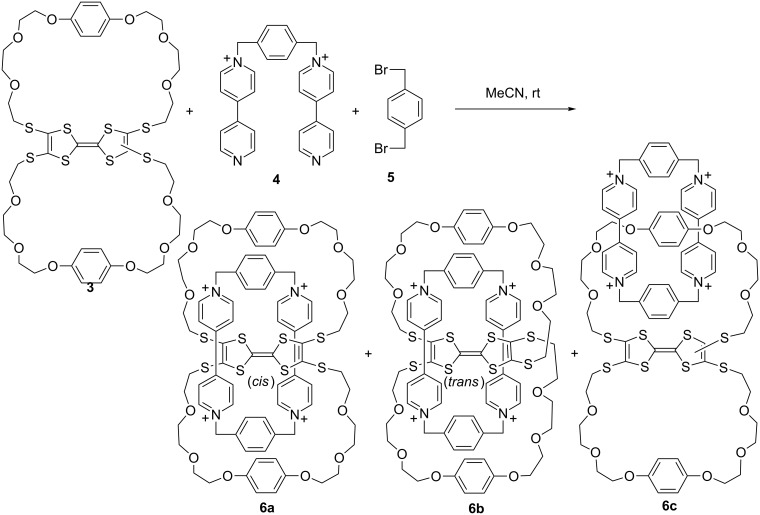
The formation of catenanes **6a–c**.

From October 1994 to December 1995, I performed postdoctoral research with Professor Jan Becher at Odense University (currently University of South Denmark) in Denmark. Through his research career, Professor Becher studied sulfur-containing molecules and since the early 1990s, tetrathiafulvalene (TTF) supramolecular chemistry. Before I went to Odense, the group had developed a very useful method of in situ generation of TTF thiolate anion from cyanoethylated precursors, which greatly simplifies the modification of the TTF core [11]. Using this method, I could prepare bimacrocycle **3** in a short time. This macrocycle was used to template the formation of the so-called tetracationic “blue box” [12–13] from **4** and **5** to give rise to the unique pseudo[3]catenanes **6a** and **6b** (Scheme 2), together with a trace amount of [2]catenane **6c** with the tetracationic cyclophane holding one of the peripheral benzene rings [14]. The TTF unit in **6a** and **6b** adopted a stable *cis* or *trans* configuration, although typically the two configurations easily isomerize into each other in solution. Macrocycle **3** is a brown solid due to the existence of the TTF unit. Catenanes **6a** and **6b** are blue as a result of the charge-transfer complex between the TTF and bipyridinium units, whereas catenane **6c** is orange, which is attributed to the charge-transfer complex between the dioxybenzene and bipyridinium units. Impressively, the three catenanes could be separated from each other using a short silica gel column. Three colorful bands could be observed from the column, which made it easy to collect the respective solutions. Another impressive occurrence was the difference in the solubility of the catenanes with different counterions in water and organic solvents. With hexafluorophosphate as the counterion, the catenanes were well-dissolved in acetonitrile, while with chloride, their solubility was poor.

My stay in Odense initiated my research in supramolecular chemistry. For many years, I maintained interest in interlocked systems. TTF is also a favorite. Many years later at Fudan, I initiated a project to study the potential of its radical cation stacking in controlling the folded conformation of linear molecules and two- and three-dimensional supramolecular polymers and frameworks. My life in the small town of Odense was also memorable. Its calm is in sharp contrast to the bustle of Shanghai where I had lived since 1987. Odense is also the hometown of Hans C. Andersen, the great Danish writer of fairy tales. Since I had read his books before, I visited the small Hans Christian Andersen Museum, which is situated in a house in the old town where he was born.

### Early research at SIOC

**Scheme 3:**
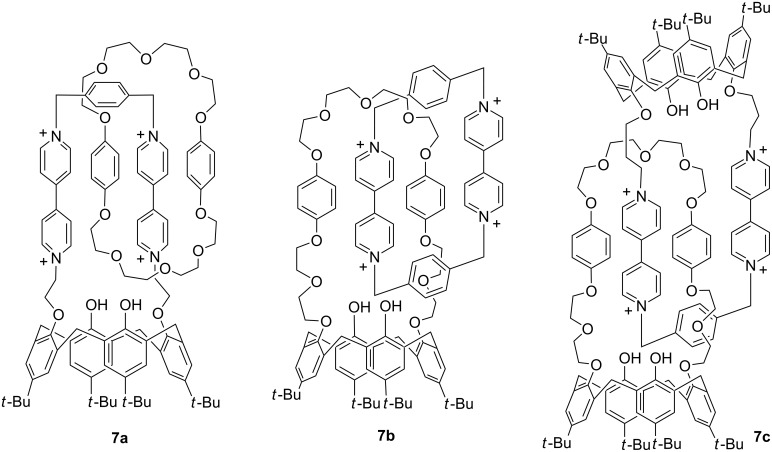
The structures of cantenanes **7a–c**.

In January 1996, I returned to SIOC. Although I had hoped to continue research in fluorine chemistry, I was assigned to work for a big contract project in the laboratory of physical-organic chemistry. After finishing the contract research, I also did a small project: constructing calix[4]arene-derived catenanes, such as **7a–c** [15] (Scheme 3). I chose this project because I believed that I could make progress in a short time. Most of the work was done by myself, but I also received assistance from several young technicians. We succeeded in investigating the effect of the calix[4]arene moiety on the relative rotation of the two rings using ^1^H NMR, and from 1998 to 2000, we published four papers from this project. However, this was generally a tough period for me. Due to some nonacademic reasons, I could not build an efficient research group after several years of effort. Therefore, I decided to make a change.

### Research at the University of Illinois

**Scheme 4:**
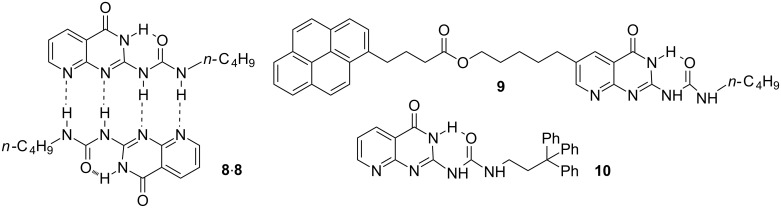
The structures of dimer **8**·**8** and compounds **9** and **10**.

In October 2000, I joined Professor Steven C. Zimmerman’s group at the University of Illinois at Urbana. The Zimmerman group had been well-known for pioneering works in hydrogen bonding-related achievements. In 1998, the group reported the extremely stable quadruple hydrogen-bonded dimers **8**·**8** [16] (Scheme 4). By using an ^1^H NMR dilution technique, a lower limit to the dimerization constant *K*_dim_ (>10^7^ M^−1^) was estimated. Professor Zimmerman hoped to make an accurate determination of the binding stability. Thus, I prepared compound **9**, which bore a pyrene unit [17] (Scheme 4), based on previously published research by Sijbesma and co-workers, who had taken advantage of the excimer signal of the pyrene dimer to evaluate the stability of their famous quadruple hydrogen bonded 2-ureido-4[1*H*]-pyrimidinone dimers [18]. The excimer exhibits an emission band at 500–600 nm, which is separated from that of the monomer. I thus determined the *K*_dim_ of **9** as 3.0 × 10^7^ M^−1^ in chloroform saturated with water ([water] ≈ 0.45 M) and 8.5 × 10^7^ M^−1^ in freshly opened chloroform ([water] ≈ 17 mM).

**Figure 1:**
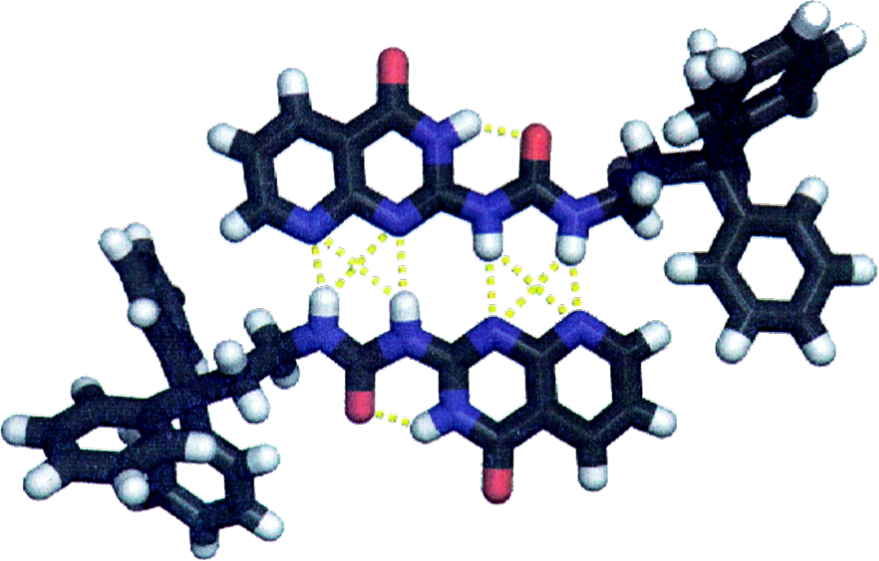
X-ray structure of **10** showing a quadruple hydrogen-bonded dimeric motif [17].

Dimer **8**·**8** had another three tautomers [16]. Thus, I also tried to obtain the single crystal structure of the binding motif. In total I prepared 28 derivatives by introducing different substituents and succeeded in growing the single crystal of compound **10** [17] (Scheme 4). The crystal structure showed the formation of a homodimer of the N(3*H*) protomer that was stabilized by four intermolecular N–H···O hydrogen bonds and the protomer itself was rigidified by an intramolecular six-membered N–H···O hydrogen bond (Figure 1). This motif was the one that had the highest proportion in chloroform.

### Research on foldamers: applications for molecular recognition and self-assembly

**Scheme 5:**
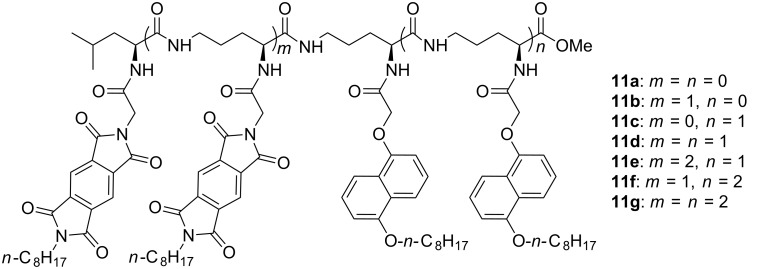
The structures of compounds **11a–g**.

**Figure 2:**
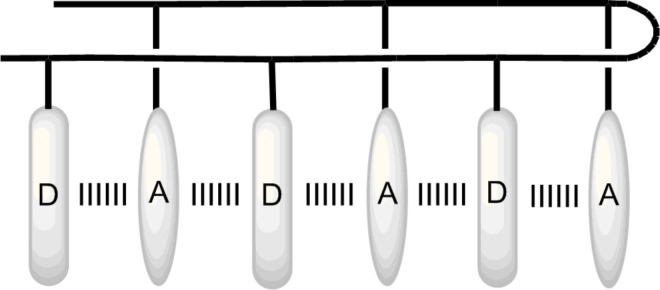
Zipper-featured folding motif of δ-peptides **11a–g** driven by the cooperative donor–acceptor interaction [22].

**Donor–acceptor interaction and π-stacking for folding.** In January 2001, I returned to SIOC again. When I left SIOC for Urbana in 2000, I had built a small research group with two graduates. Thanks to the persistence of Professor Xi-Kui Jiang [19–20], one of the greatest physical organic chemists in China, my small group survived until I returned to the institute. I was so impressed by the work of Professor Zimmerman on the folding of linear urea derivatives driven by intramolecular hydrogen bonding [21], that in 2001, our group started several projects searching for new folded frameworks. All the projects were done by Ph.D. students. Xin Zhao, who is currently a professor at SIOC, finished the first project in 2004. He prepared L-ornithine-derived δ-peptides **11a–g**, which bore one to three electron-deficient pyromellitic diimide (PDI) and electron-rich 1,5-dioxynaphthalene (DAN) units on the two sides of the backbones [22] (Scheme 5). An intramolecular donor–acceptor interaction between the DAN and PDI units, which is a well-established noncovalent force [23–24], induced the backbones to fold into zipper-featured foldamers in less polar chloroform and polar DMF (Figure 2). This finding was supported by ^1^H NMR, UV–vis, and fluorescence quenching studies. As expected, the folding state became more compact for longer sequences, which possess more donor–acceptor interacting sites. UV–vis experiments indicated that the folding state remained, even at 150 °C in DMF.

**Scheme 6:**
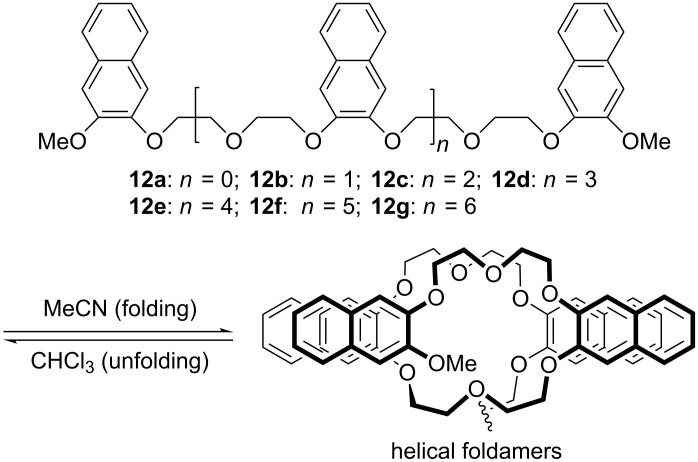
The structures of compounds **12a–g** and the formation of the helical conformation by the longer oligomers.

Encouraged by the above work, Junli Hou, who is currently a professor at Fudan University, prepared naphthalene-incorporated oligo(ethylene glycols) **12a–g** [25] (Scheme 6). UV–vis, ^1^H NMR, and fluorescence experiments in chloroform–acetonitrile binary solvents revealed that the naphthalene units in longer **12f–h** stacked intramolecularly to induce the oligomeric chains to form a helical conformation at high acetonitrile content (Scheme 6). The compact helical conformation gave rise to a cavity similar to that of 18-crown-6 and thus could complex ammonium or ethane-1,2-diaminium in acetonitrile. The stability of the complexes increased with the elongation of the ethylene glycol chains.

**Scheme 7:**
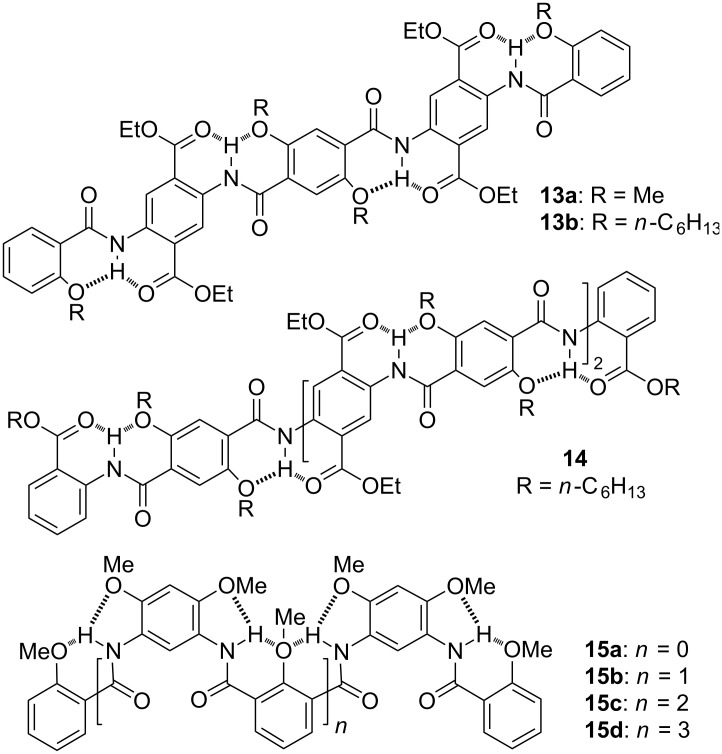
The structures of compounds **13a,b**, **14**, and **15a–d**.

**Aromatic amide oligomers: extended secondary structures.** Our students also tried to make use of hydrogen bonding to control the conformation of aromatic amide backbones. Previously, Hamilton [26], Gong [27], Huc and Lehn [28], and Huc [29] had developed several series of elegant folded frameworks. We thus focused on the creation of extended frameworks, which we expected to be useful for the design of functional materials [30]. In 2004, Zongquan Wu, who is currently a professor at Hefei University of Technology, reported the formation of straight conformations by oligomers **13a,b** and **14**, which was driven by successive intramolecular hydrogen bonding [31] (Scheme 7), whereas Jiang Zhu, who is currently an associate professor at North Sichuan Medical College, described the zigzag conformation of oligomers **15a–d**, which is also stabilized by intramolecular hydrogen bonding [32] (Scheme 7).

**Scheme 8:**
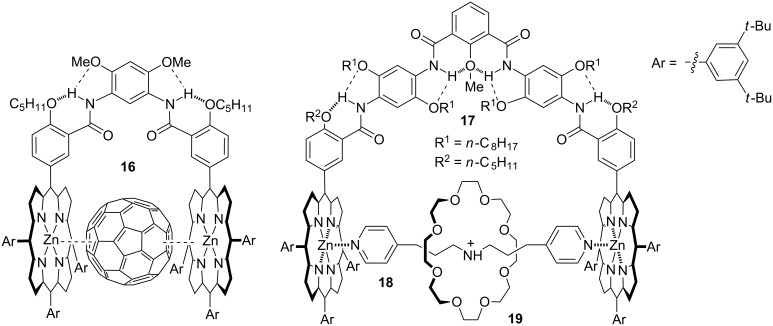
The structures of complex C_60_ 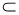 **16** and dynamic [2]catenane formed by compounds **17–19**.

The aromatic units in oligomers **13–15** can be easily combined into one sequence. By changing their number and position, sequences of different length and shape or in a controlled conformation can be designed [33–34]. Thus, oligomers **13–15** may be considered as structural prototypes for creating new modifiable backbones. For example, porphyrin-appended U-shaped molecular tweezers **16** and **17** have been produced (Scheme 8). Compound **16** complexed C_60_ or C_70_ or their derivatives in chloroform or toluene through porphyrin–C_60_ stacking [35], while compound **17** strongly complexed **18** in the chloroform–acetonitrile binary medium [36] (Scheme 8). Because **18** could further form a threaded complex with 24-crown-8 **19** driven by multiple O···H–N hydrogen bonds, the three components self-assembled into a unique dynamic [2]catenane. Under low temperature, this dynamic [2]catenane could be quantitatively generated. The intramolecular hydrogen bonds formed by the aromatic amide linkers remarkably enhanced the complexation of the porphyrin units towards the fullerene and the bipyridine ligand.

**Scheme 9:**
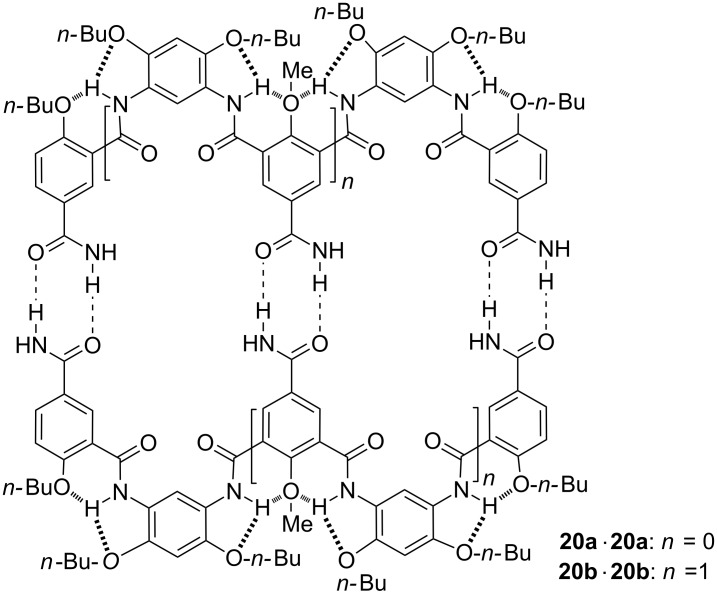
The structure of homodimers **20a·20a** and **20b·20b**.

Jiang further introduced amide subunits to the *para*-position of the ether groups of the benzamide rings of oligomers **15a** and **15b** to produce **20a** and **20b** [37], respectively (Scheme 9). ^1^H NMR dilution experiments in chloroform-*d* revealed that both compounds formed stable homodimers **20a·20a** and **20b·20b**, with *K*_dim_ being 3.0 × 10^3^ and 2.3 × 10^5^ M^−1^, respectively. In contrast, even in nonpolar benzene-*d*_6_, the *K*_dim_ for the dimerization of benzamide was only 40 M^−1^. The result again shows that the intramolecular hydrogen bonding of the aromatic amide backbones promoted the appended amide subunits to bind in a cooperative manner by preorganizing the backbones.

**Scheme 10:**
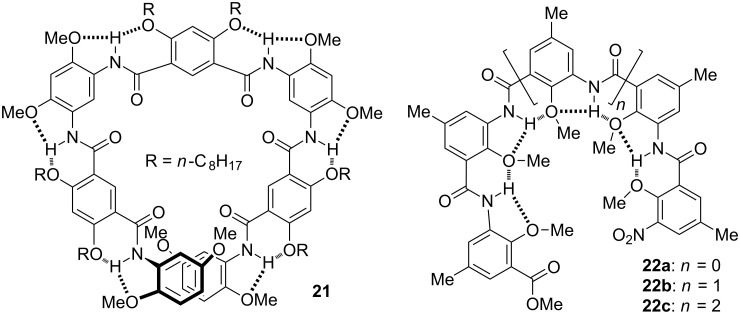
The structures of foldamers **21** and **22a–c**.

**Aromatic amide oligomers: folded and helical secondary structures.** In 2005, Huiping Yi finished the synthesis and characterization of aromatic amide oligomers **21** and **22a–c** [38–39] (Scheme 10). These two series of oligomers folded into helical secondary structures driven by intramolecular hydrogen bonding, which is typical for aromatic amide backbones [40–42]. The diamine and diacyl chloride precursors for **21** had been used by Gong and co-workers to prepare the first family of hydrogen bonding-promoted aromatic amide macrocycles [43]. The main aim for designing these folded structures was to explore their potential functions as acyclic receptors. Moore et al. had utilized this approach to investigate the binding of *m*-phenylene ethynylene foldamers for nonpolar organic molecules in polar media [44]. All the C=O oxygen atoms of **21** point into the cavity of the helix, which has a diameter of approximately 0.8 nm. Thus, **21** complexed alkylated saccharide derivatives and a guest with three hydroxyl groups in chloroform. The binding also induced the backbone of **21** to produce helicity bias [38]. The methoxy groups of **22a–c** are all located inwards. These oxygen atoms are potential hydrogen bonding acceptors. ^1^H NMR and fluorescence experiments in chloroform showed that this series of foldamers complexes primary and secondary alkyl ammonium products [39].

**Scheme 11:**
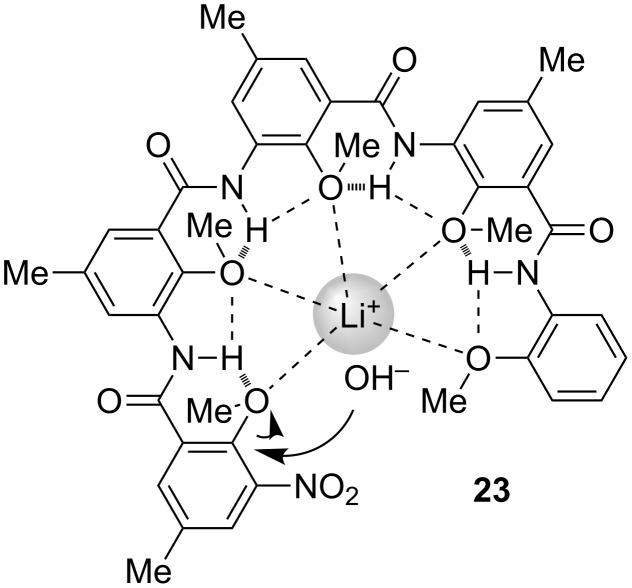
Complexation-promoted hydrolysis of foldamer **23**.

In most cases, we investigated the binding of foldamers for different guests in less polar chloroform. However, during the synthesis of the **22** series, which involved the hydrolysis of the methyl ester at one end with lithium hydroxide in heated dioxane–water solution, Huiping found that the nitro-bearing anisole unit at the other end was also hydrolyzed to afford a phenol derivative [45]. In contrast, a short control did not exhibit similar reactivity. These observations suggested that the folded conformation, such as in **23**, promoted the hydrolysis of the anisole, which we ascribed to the complexation of the foldamer to Li^+^ cation (Scheme 11). We proposed that this complexation increased the efficient concentration of the OH^−^ anion and also activated the nitro-bearing anisole. This result supports that the intramolecular MeO···H–N hydrogen bonding worked even in a highly polar solvent. The theoretical study by Pophristic and co-workers also shows that this hydrogen bonding exists, to some extent, in polar aqueous environments [46]. The high stability of the folded conformation of the **22** series also make them useful frameworks for creating a variety of complicated supramolecular architectures [42].

**Scheme 12:**
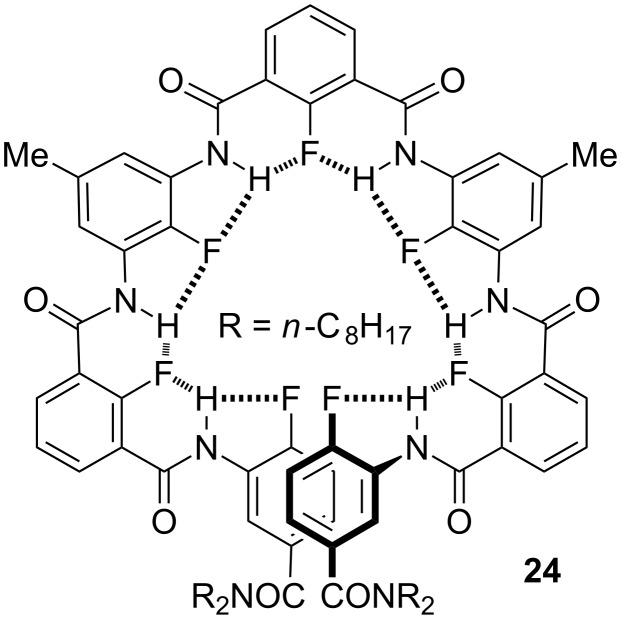
The structure of foldamer **24**.

Although alkoxyl groups had been popularly used as acceptors for intramolecular hydrogen bonding [40–42], I was also interested in looking for other substitutes. For this purpose, I envisioned fluorine might be a good candidate, given its highest electronegativity. An investigation by Dunitz and Taylor led to the conclusion that “organic fluorine hardly ever accepts hydrogen bonds, that is, it does so only in the absence of a better acceptor” [47–48]. We conjectured that fluorine might work as an acceptor for intramolecular five- and six-membered F···H–N hydrogen bonding for aromatic amides because of their co-planarity. Chuang Li thus prepared oligomer **24** and shorter analogues [9]. Systematic ^1^H NMR experiments in chloroform-*d* and crystal structure analysis of model molecules all supported that fluorine was engaged in the expected intramolecular F···H–N hydrogen bonding and **24** formed a helical conformation (Scheme 12). Moreover, Chuang found that intermolecular F···H–N hydrogen bonding could also be formed between **23** and aliphatic ammonium and, at high concentrations in chloroform, a chiral aliphatic ammonium induced **23** to produce helicity bias.

One important difference between fluorine and alkoxy groups is that fluorine does not cause a steric effect on aromatic stacking. Zeng and co-workers demonstrated this difference by crystallizing a fluorine-bearing pentagon macrocycle [49–51]. No stacking was observed for the methoxy-derived pentagon macrocycle. In contrast, their fluorine-engaged pentagon macrocycle stacked strongly. Moreover, the macrocycle stacked in the 2D space to give rise to the mathematically predicted, most densely packed lattice for a C_5_-symmetric macrocycle [49].

Jiang and co-workers also prepared fluorine-containing quinoline oligoamides, which they found self-assembled into unique double and quadruple helices [52–53]. The fluorine atoms in the oligomers induced the backbone to fold by forming intramolecular F···H–N hydrogen bonding, whereas the small size of fluorine allowed the folded sequences to stack into double and quadruple helices. In an elegant study, they prepared fluorine-containing hybrid sequences with different aromatic subunits, whose helical states could hold one linear molecule to give rise to foldamer-derived dynamic rotaxanes [54].

**Scheme 13:**
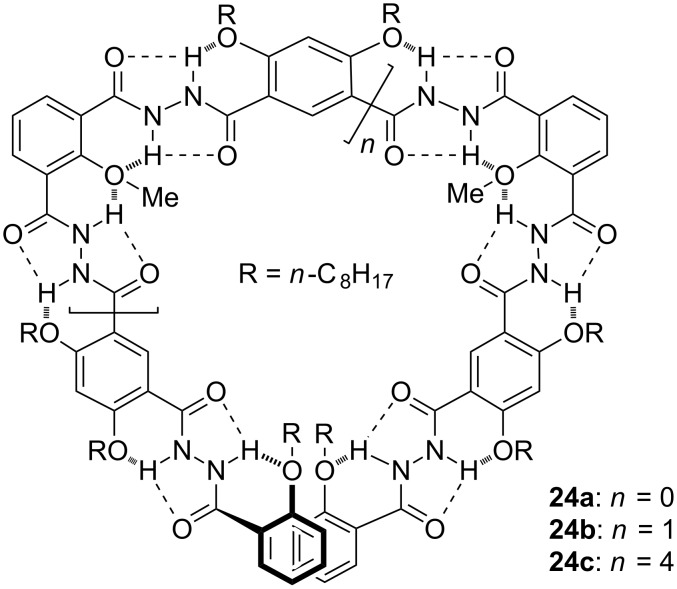
The structures of foldamers **24a–c**.

**Aromatic hydrazide foldamers:** Aromatic hydrazides have a high propensity towards co-planarity [55–56]. Nowick introduced this unit into peptide backbones to increase the stability of artificial β-sheets [55]. Junli hoped to extend the backbones of hydrogen-bonded foldamers and thus prepared oligomers **24a–c** [57] (Scheme 13). The octoxy groups provided solubility in common solvents such as chloroform. These and the methoxy groups formed successive hydrogen bonds to induce the backbones to form folded conformations, which have a cavity of about 1 nm in diameter. These folded structures could host alkylated saccharides in chloroform, which was stabilized by intermolecular hydrogen bonding formed between the carbonyl oxygen atoms of the foldamers and the hydroxy groups of the saccharides. By changing the position of the alkoxy groups, the shape of the backbones can be readily tuned. The large cavity tolerates the existence of the methoxy groups for the backbones to stack efficiently. Thus, the backbones can stack to form vesicles or gelate organic solvents depending on the appended side chains [42]. Jiang and co-workers prepared methoxy-free backbones [58]. It is expected that these backbones should exhibit increased conformational flexibility. However, they still displayed quite strong binding capacity to anions and saccharides.

**Scheme 14:**
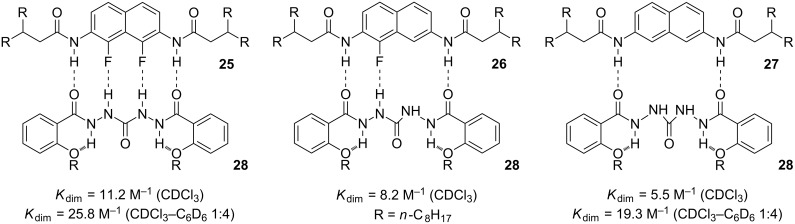
Proposed structures of heterodimers **25·28**, **26·28**, and **27·28**.

**Studies on N–H····X (X = F, Cl, Br, I) hydrogen bonding.** At SIOC, our group is the only one dedicated to physical-organic chemistry. Thus, we maintained a longstanding interest in the fundamental aspects of noncovalent forces. The establishment of intramolecular N–H····F hydrogen bonds raised the possibility of quantitative assessment of intermolecular N–H····F hydrogen bonds. Since this hydrogen bonding is generally weak, Yanhua Liu prepared compounds **25–27** [59] (Scheme 14), which have the identical backbones, in order to evaluate the stability of the heterodimers formed between them and **28** [56]. In chloroform, *K*_dim_ for the three complexes was determined to be 11.2, 8.2 and 5.5 M^−1^, respectively. In less polar binary chloroform–benzene (1:4, v/v), the *K*_dim_ values of the first and third complexes were increased significantly to 25.8 and 19.3 M^−1^, respectively. These results indicate that the intermolecular N–H····F hydrogen bond did exist for aromatic derivatives, but is quite weak.

After the establishment of the intramolecular N–H····F hydrogen bond motif, we further prepared various model molecules to exploit the possibility of forming similar N–H····X (X = Cl, Br, I) hydrogen bonding motifs by aromatic amide derivatives [60]. We found that all these halogen atoms are able to form this hydrogen bonding, but the stability decreases successively, which is consistent with the decrease of their electronegativity, but may also reflect the increase of the van der Waals radius. These weak intramolecular hydrogen bonds cannot compete with the strong intermolecular N–H···O=C hydrogen bonds of the amide groups. Thus, for their formation, the latter has to be suppressed. For the same halogen atom, the five-membered N–H····X (X = Cl, Br, I) hydrogen bond is generally easier to form than the six-membered one. One straightforward explanation for this difference is that the formation of the former would confine the rotation of one single bond, while for the latter, it would confine two single bonds. Jiang and Huc and co-workers successfully utilized the five-membered N–H···Cl hydrogen bond to construct quinoline amide-derived double helices [53].

**Scheme 15:**
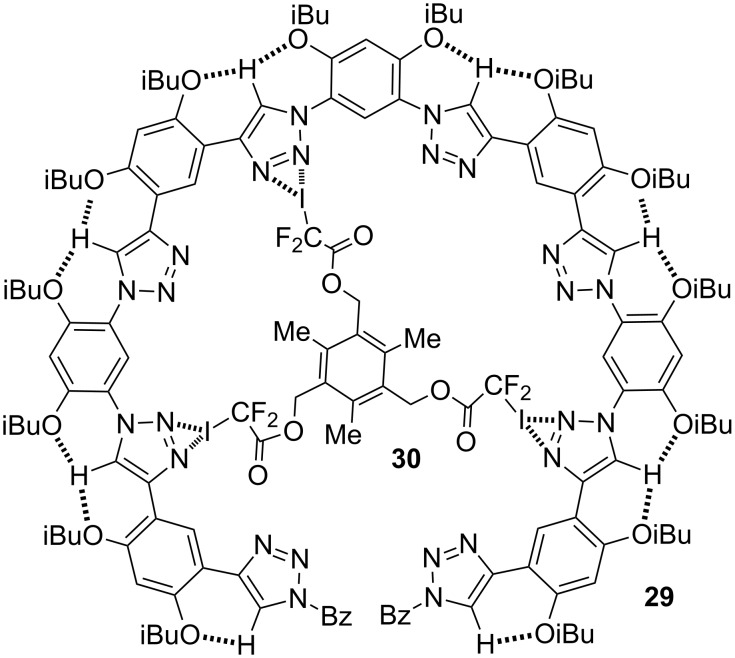
Proposed structure of complex formed by **29** and **30**.

**C–H····X (X = OR, F) hydrogen bonding-driven 1,2,3-triazole foldamers.** In 2008, several groups independently described that the intermolecular C–H····Cl^−^ hydrogen bonding could induce benzene-linked 1,2,3-triazole oligomers to form folded or helical conformations [61–63]. Currently, this family of foldamers have found wide applications in anion binding and design of photo-active molecular devices [64–66]. We were interested in developing inherently folded structural patterns for aromatic oligotriazole backbones. In 2009, Yuanyuan Zhu established that 1,5-diphenyl-1,2,3-triazole formed an intramolecular six-membered C–H····OMe hydrogen bonds [67]. In 2012, Beye Lu further demonstrated that fluorine, chlorine or even bromine could form similar intramolecular C–H····X hydrogen bonds [68]. Using the C–H····O hydrogen bonding motif, Liyan You created a family of oligotriazole foldamers, including **29** (Scheme 15), which had a cavity of approximately 1.8 nm in diameter [69]. At first we expected that this series of triazole foldamers would be good hydrogen bonding acceptors. Intriguingly, the foldamers did not exhibit observable binding affinity to saccharides or amide derivatives even in less polar chloroform. Liyan designed different molecules to evaluate their binding to these triazole foldamers. He finally found that these foldamers were good halogen bonding receptors for tritopic and ditopic guests, including **30** in dichloromethane or its mixture with hydrocarbons.

Yanhua further utilized the C–H····F hydrogen bonding to induce the folding of the same series of benzene/triazole oligomers [10]. Recently, Jiang and co-workers reported that when fluorine or chlorine was introduced to the 2-position of the *meta*-substituted benzene linkers, the corresponding triazole oligomers folded to give new foldamers, which were stabilized by successive intramolecular C–H····F or C–H····Cl hydrogen bonds [70].

**Scheme 16:**
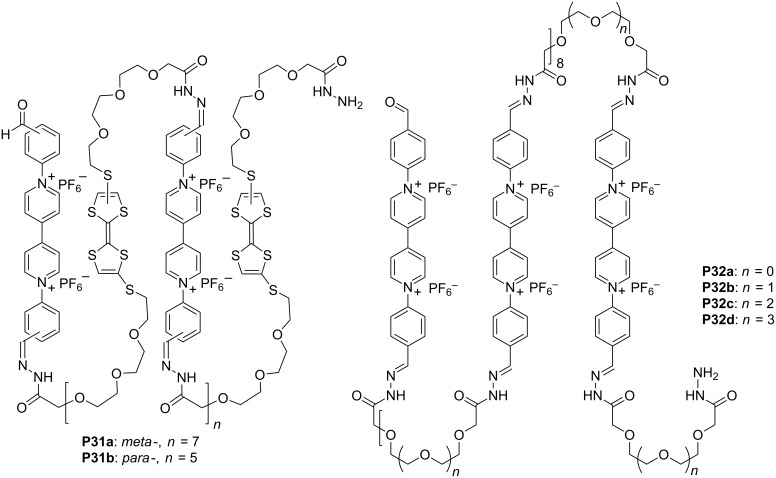
The structures of polymers **P31a,b** and **P32a–d**.

**Conjugated radical cation dimerization-driven pleated foldamers.** The stacking of the radical cations of viologen or TTF were observed in 1964 and 1979 [71–72]. This stacking is typically weak. Several approaches have been developed to enhance this stacking [73–76]. As a result, strong stacking, which leads to the formation of stable homodimers, has been observed in many elegantly designed molecules and supramolecules. In recent years, this stacking has been utilized as a tunable, noncovalent force to induce the formation of interlocked systems and molecular switches [77–78]. Our longstanding interest in foldamers prompted us to explore the application of this noncovalent force for the generation of new, folded patterns. Thus, Lan Chen prepared polymers **P31a** and **P31b** from the corresponding dialdehyde and di(acylhydrazine) precursors by forming dynamic hydrazone bonds [79] (Scheme 16). This dynamic covalent chemistry approach allowed for quick synthesis of viologen/TTF-alternating polymers. Driven by the intramolecular donor–acceptor interaction between the TTF and viologen units, the polymers folded into pleated conformations in acetonitrile. Upon oxidation of the TTF units to radical cation TTF**^·+^**, the polymers adopted flexible conformations. When the viologen units were reduced to radical cations, the radical cations stacked intramolecularly to induce the backbone to form another kind of pleated secondary structure. Yunchang Zhang further illustrated that upon reducing the viologen units into radical cations, their intramolecular stacking also induced polymers **P32a–d** to form pleated conformations [80] (Scheme 16). This occurred despite the fact that the folding of **P32a** needed the assistance of alkaline metal ions like Li^+^.

### Solution-phase supramolecular metal–organic frameworks (MOFs)

**Scheme 17:**
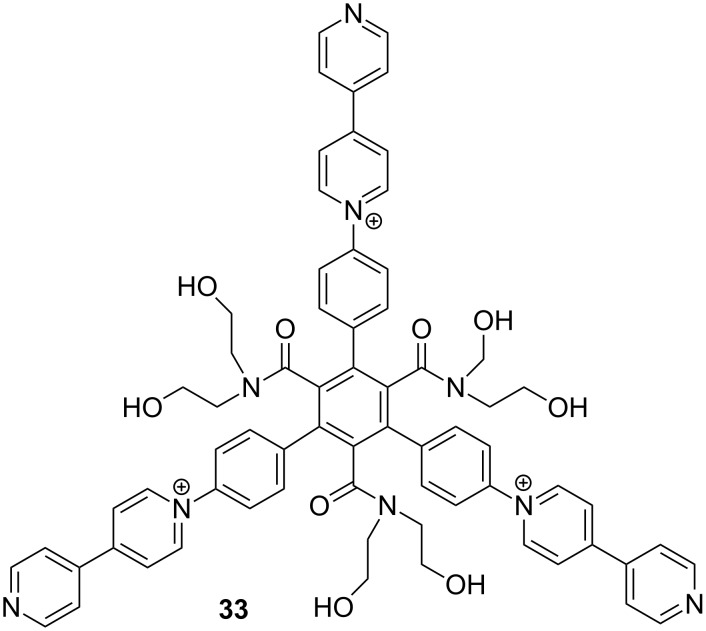
The structure of compound **33**.

Periodicity is the key feature of single crystals in which molecules arrange repeatedly in the three-dimensional space. Porous crystals such as metal–organic frameworks (MOFs) exhibit many unique properties mainly due to their large surface area. However, the achievement of periodicity is a challenge in solution for self-assembled architectures as a result of the impact of the solvent and the weakness of noncovalent forces that hold monomeric components together. In early 2012, I discussed the initiation of a project with Kangda Zhang to explore the possibility of generating honeycomb, supramolecular frameworks in water. In a short time, he prepared one triangular target molecule. However, the molecule was poorly soluble in water. He then prepared compound **33** [81] by introducing three hydrophilic amide chains, which provided good solubility in water (Scheme 17). Shortly after, Kangda revealed that the three phenyl-bipyridine units strongly stacked in the two-dimensional space when mixing with 1.5 equiv of cucurbit[8]uril (CB[8]), which could encapsulate the stacked bipyridine dimers [82–83]. In collaboration with Yi Liu at Lawrence Berkeley National Laboratory in the United States, we conducted solution-phase, small-angle X-ray scattering synchrotron experiments on the mixture of **33** and CB[8] in water (1:1.5, 3.0 mg/mL), which supported the periodicity of the 2D honeycomb SOF structures in solution. Liang Zhang found that the stacking of viologen radical cations could also drive a similar triangular molecule to form 2D SOF, which was further stabilized with CB[8] by encapsulating the stacking radical cation dimer [84]. Very recently, Zhao and co-workers reported that a donor–acceptor interaction could drive the formation of square-shaped 2D MOFs from a porphyrin-derived bipyridinium precursor and 2,6-dioxynaphthalene-derived ditopic precursor [85]. In this case, the 2,6-dioxynaphthalene–dipyridinium donor–acceptor complex was also stabilized by CB[8]. These results showed that generation of 2D SOFs can be induced by discrete noncovalent forces.

**Scheme 18:**
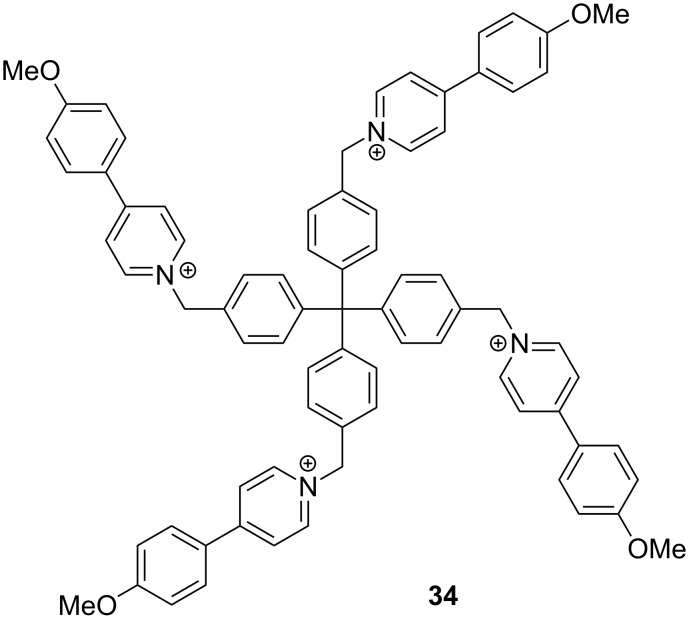
The structure of compound **34**.

In 2014, Jia Tian further extended the concept of solution-phase SOF to 3D space from the self-assembly of tetrahedral **34** and CB[8] [86] (Scheme 18). The 1:2 mixture in water generated a 3D, homogeneous SOF of diamond topology. The pores of the 3D SOF could be observed by high-resolution TEM. As a supramolecular “ion sponge”, the framework adsorbed various anionic guests, including drugs, peptides and DNA. These new 2D and 3D homogeneous SOFs all have defined cavities or pores, which are expected to display new, interesting properties [87].

### Future perspectives

My research on foldamers was heavily affected by the work of Professor Xi-Kui Jiang on the self-coiling of organic molecules in aqueous media driven by hydrophobicity [19]. At an early stage, we developed quite a number of folding patterns to explore the so-called functions. Finally, I established the hydrogen-bonded aromatic amide and hydrazide sequences as a longstanding research area. With these sequences as a structural basis, we were able to investigate many interesting phenomena or concepts in supramolecular chemistry such as stimuli responsiveness, “sergeant–soldier effect”, and supramolecular devices [88]. It is also noteworthy that the preorganization feature of the amide sequences can remarkably increase the selectivity of macrocyclization [42,51,89]. By making use of the dynamic covalent chemistry approach, we have demonstrated that complicated macrocycles can be obtained in nearly quantitative yields [90].

My research on aromatic foldamers has lasted for more than ten years. In collaboration with Junli, we recently found that the tubular cavity of hydrazide foldamers could mediate the transmembrane transport of K^+^ [91]. One ongoing project is to explore the routes for improving the transport selectivity for proton, cations and anions by making use of helical foldamers as channels. Long helical foldamers can theoretically form deep spring-shaped tubes. However, the synthesis and characterization of long foldamer polymers has been a challenge [92–94]. I hope that we can find solutions to address this issue in the future. Particularly, we are interested in obtaining stable, single macromolecular tubes, probably after suitable postmodification. Solution-phase SOFs are new, homogeneous, porous architectures. As ordered supramolecular polymeric electrolytes, this family of self-assembled systems may be developed as useful adsorbing materials. Currently we are investigating new behaviors of molecules or macromolecules adsorbed by SOFs in solution.
